# microRNA-34a overexpression inhibits cell migration and invasion via regulating SIRT1 in hepatocellular carcinoma

**DOI:** 10.3892/ol.2019.11048

**Published:** 2019-11-04

**Authors:** Jianhui Zhou, Wenying Zhou, Fangen Kong, Xiaoyu Xiao, Haoyu Kuang, Yingxian Zhu

Oncol Lett 14: 6950-6954, 2017; DOI: 10.3892/ol.2017.7090

Following the publication of the above article, an interested reader drew to our attention the fact that, based on the sequence of the GAPDH primer reported in Table I, the authors had apparently performed their experiments with a primer for GAPDH that belonged to a mouse, whereas the cell lines used in this paper were human cell lines. Secondly, the primary data were not included in the paper for the cell migration experiments (Fig. 2), and issues were also raised concerning the presentation of the data in the western blots featured in Fig. 4.

Upon enquiring with the authors about these matters, they explained that the primer sequence information was presented incorrectly in Table I, although the correct primers had in fact been used for this study; the correct primer sequences as they should have been presented are shown in the new version of Table I opposite. They were unable to consult their original data for Figs. 2 and 4, and so these experiments have been re-performed; the revised version of Fig. 2 is shown opposite, and that of Fig. 4 is on the subsequent page. The results derived from these repeated experiments concurred with the results and the conclusions already reported in this study. The authors wish to thank the reader for drawing these issues to their attention, and apologize to the Editor and to the readership for any inconvenience caused.

## Figures and Tables

**Figure 2.**
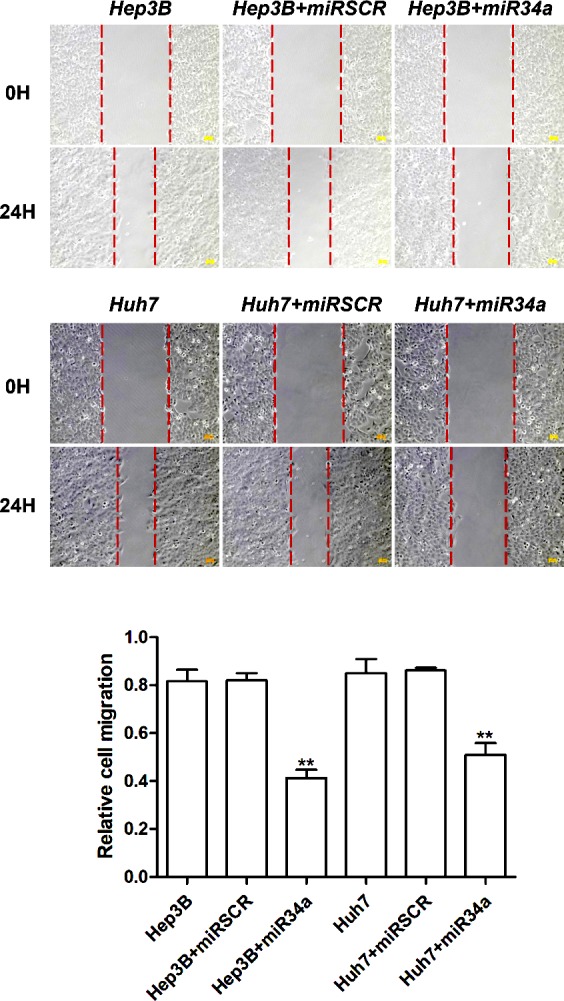
Effects of miR-34a on hepatocellular cell migration. Compared with the control cells, overexpression of miR-34a significantly decreased the number of migrated Hep3B and Huh7 cells. Images representing the extent of cellular migration for the different groups at 0 h and after 24 h of incubation are shown, and the quantification of these data is shown in the bar chart. **P<0.01 vs. control cells. miR, microRNA; miRSCR, scramble miR-34a.

**Figure 4.**
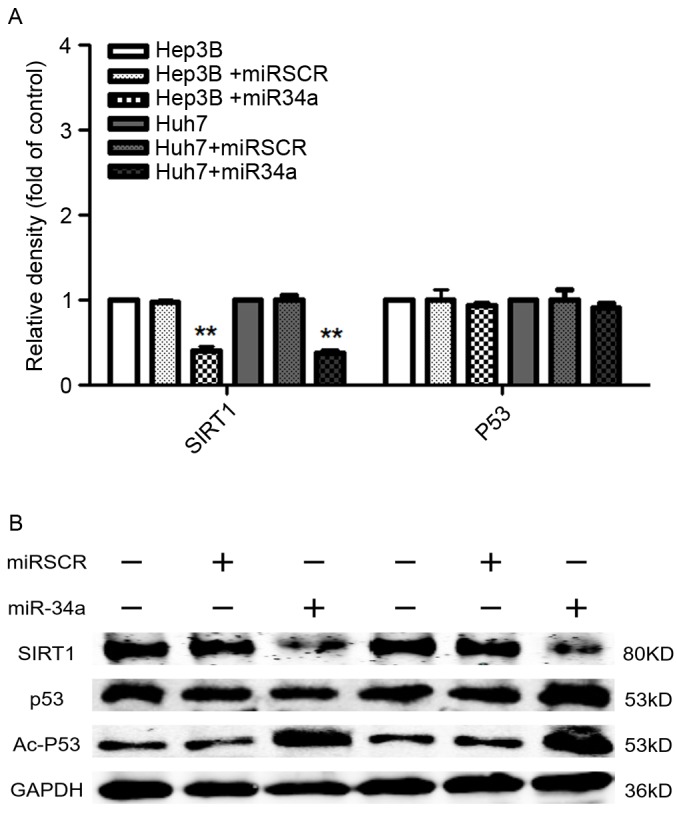
Effects of miR-34a expression on cell metastasis-associated protein expression in hepatocellular cells. (A) Overexpression of miR-34a signifi- cantly decreased the mRNA levels of SIRT1. (B) Overexpression of miR-34a decreased the protein expression of SIRT1, but increased the expression of Ac-p53. **P<0.01 vs. control cells. miR, microRNA; miRSCR, scramble miR-34a; SIRT, sirtuin; p53, tumor protein 53, Ac-p53, acetylated p53.

**Table I.** Primers used for target amplification in the present study.

Name	Primer	Sequence (5′-3′)
GAPDH	Sense	AGCCACATCGCTCAGACAC
	Antisense	GCCCAATACGACCAAATCC
SIRT1	Sense	GCTTATTTGTCAGAGTTCCCACCC
	Antisense	CAGCATTTTCTCACTGTTCCAGCC
p53	Sense	GAGGTTGGCTCTGACTGTACC
	Antisense	TCCGTCCCAGTAGATTACCAC

SIRT, sirtuin; p53, tumor protein 53.

